# In Response to Nguyen et al: Mold Active Triazole Prophylaxis Utilization among Patients with Acute Myeloid Leukemia in a Community Hospital

**DOI:** 10.1093/ofid/ofae135

**Published:** 2024-03-08

**Authors:** Michael J Williams, Margaret Cook, Brittany Mejaki, Brian P Buggy

**Affiliations:** Department of Pharmacy, Aurora St Luke's Medical Center, Milwaukee, Wisconsin, USA; Department of Pharmacy, Aurora St Luke's Medical Center, Milwaukee, Wisconsin, USA; Department of Pharmacy, Aurora St Luke's Medical Center, Milwaukee, Wisconsin, USA; Infectious Diseases Section, Aurora St Luke's Medical Center, Milwaukee, Wisconsin, USA

To the Editor—From the perspective of a large community hospital, we appreciate the publication of real-world data regarding the utility of mold-active triazoles (MATs) in limiting the development of invasive fungal infections among high-risk patients [1]. However, despite reporting patients’ primary insurance, we did not see mentioned the prescription cost to patients. This concern of cost-effectiveness has previously been highlighted on a systematic level in a report that evaluated both the increasing use and wholesale acquisition cost of MATs specifically among Medicare Part D enrollees [2]. Inspired by Nguyen et al, we sought to investigate our own MAT utilization.

We retrospectively identified patients who were newly diagnosed with acute myeloid leukemia (AML) and began inpatient chemotherapy between 1 January 2023 and 16 August 2023. The primary aim was to identify MAT utilization, of which posaconazole is preferred [3], during the initial hospital stay and at discharge. Secondary aims included identifying reasons for non-MAT utilization and financial barriers to outpatient MAT continuation via pharmacist-generated test claims.

Twenty-eight patients, with a median age of 67 years (range, 34–81 years), were evaluated. Insurance types were Medicare (53.5%), private (35.7%), Medicaid (7.1%), and none (3.7%). Posaconazole was the initial therapy in 53.6% followed by fluconazole (35.7%), micafungin (7.1%), and amphotericin (3.6%) (Figure 1). Reasons for alternative agents included significant (category X) interactions with concurrent medications (n = 2), hepatic dysfunction (n = 2), and physician decision/other (n = 9).

**Figure 1.**
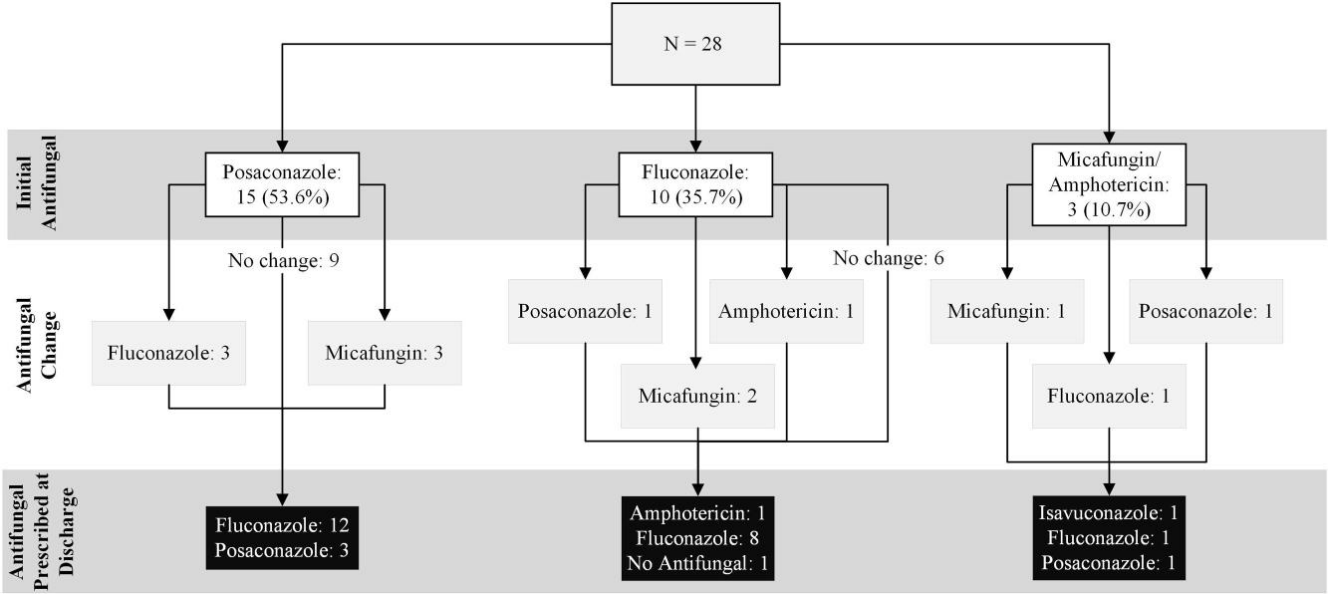
Illustration of initial, sequenced, and discharging antifungal selection.

Thirteen patients (46.4%) experienced a change in antifungal therapy during their admission (Figure 1). Six were on posaconazole but sequenced to a non-MAT. The median duration of posaconazole prior to change was 11 days (range, 4–21 days). Reasons included elevated transaminases, QTc prolongation, or known cost barriers. Two patients were sequenced from a different antifungal to posaconazole.

At discharge, 75% received fluconazole (Figure 1). Posaconazole or isavuconazole was dispensed for 5 patients (17.9%). One continued outpatient amphotericin and another did not require continuation. Reasons for discharging on non-MATs were elevated transaminases (n = 2), insurance denials due to drug interaction (n = 2), copays (n = 8), MAT coverage not checked (n = 7), or other/unknown (n = 2). No test claims for posaconazole were performed for patients who never received posaconazole.

Of 17 patients who received posaconazole at any time, all had prescription coverage assessed. Prior authorizations were completed and approved for 8 of 9 Medicare patients (1 was out of network). The average initial Medicare copay per month was $1137 (range, $476–$1523), thus prompting a cost-related change for all Medicare cardholders. Medicaid or private insurance average copays were $0 or $35.63 (range, $7.50–$65), respectively. Isavuconazole coverage was examined in 2 cases: $0 (Medicaid) and $1200 (private). Voriconazole was not investigated for any patient per provider discretion.

While our data are a small sampling compared to the original report, the nearly 40 times higher average copay per month among Medicare patients versus other payors combined is startling. Excluding 5 patients who were able to continue posaconazole beyond the inpatient stay, our median 11-day duration of posaconazole among patients who had to switch therapies pales in comparison to the median 46 days reported by the authors (66 days for any MAT) [1]. We are intrigued by their findings and wonder if patients remained in-hospital, were able to fill outpatient prescriptions, or received assistance from the project's financial supporters. Stratification of these data would aid in guiding clinicians on the degree of real-world MAT use.

Limitations of our review include the lack of MAT coverage investigation for patients who never received an MAT. Regardless, we expect this reflects the population as a whole as Medicare patients exclusively could not receive a MAT at discharge. It is not known to the authors if resources for copay support were explored (eg, discount cards or manufacturer assistance). However, government-funded programs like Medicare are often excluded from participation, thus significantly limiting options for affordability.

Despite not formally assessing patient outcomes as an aim, we observed 1 breakthrough aspergillosis infection on fluconazole. We note the higher incidence of breakthrough fungal infections reported by Nguyen et al [1] in patients who received sequenced therapies (15.8%) compared to single MAT alone. Nearly half of our population experienced a change in antifungal, most frequently to a non-MAT agent.

In a population where disease treatment options are often costly, it is unfortunate that we find supportive care agents to also be price prohibitive. Our findings have led us to actively seek MAT coverage at the time of AML diagnosis. We fully support clinical MAT use in these high-risk patients as described by Nguyen et al [1], but until copays are reduced, universal out-of-hospital MAT prophylaxis in appropriate patients will remain challenging.

## Notes

***Patient consent.*** This project did not include factors necessitating patient consent.
